# Complete genome sequence of *Marinobacter adhaerens* type strain (HP15), a diatom-interacting marine microorganism

**DOI:** 10.4056/sigs.922139

**Published:** 2010-09-28

**Authors:** Astrid Gärdes, Eva Kaeppel, Aamir Shehzad, Shalin Seebah, Hanno Teeling, Pablo Yarza, Frank Oliver Glöckner, Hans-Peter Grossart, Matthias S. Ullrich

**Affiliations:** 1 Jacobs University Bremen, School of Engineering and Science, Bremen, Germany; 2 Max Planck Institute for Marine Microbiology, Microbial Genomics and Bioinformatics Group, Bremen, Germany; 3 Institut Mediterrani d'Estudis Avançats, Marine Microbiology Group, Esporles, Spain; 4 IGB-Neuglobsow, Dept. Limnology of Stratified Lakes, Stechlin, Germany

**Keywords:** marine heterotrophic bacteria, diatoms, attachment, marine aggregate formation

## Abstract

*Marinobacter adhaerens* HP15 is the type strain of a newly identified marine species, which is phylogenetically related to *M. flavimaris*, *M. algicola*, and *M. aquaeolei*. It is of special interest for research on marine aggregate formation because it showed specific attachment to diatom cells. *In vitro* it led to exopolymer formation and aggregation of these algal cells to form marine snow particles. *M. adhaerens* HP15 is a free-living, motile, rod-shaped, Gram-negative gammaproteobacterium, which was originally isolated from marine particles sampled in the German Wadden Sea. *M. adhaerens* HP15 grows heterotrophically on various media, is easy to access genetically, and serves as a model organism to investigate the cellular and molecular interactions with the diatom *Thalassiosira weissflogii*. Here we describe the complete and annotated genome sequence of *M. adhaerens* HP15 as well as some details on flagella-associated genes. *M. adhaerens* HP15 possesses three replicons; the chromosome comprises 4,422,725 bp and codes for 4,180 protein-coding genes, 51 tRNAs and three rRNA operons, while the two circular plasmids are ~187 kb and ~42 kb in size and contain 178 and 52 protein-coding genes, respectively.

## Introduction

Strain HP15 (DSM 23420) is the type strain of the newly established species *Marinobacter adhaerens* sp. nov. and represents one of 27 species currently assigned to the genus *Marinobacter* [1]. Strain HP15 was first described by Grossart *et al.* in 2004 [2] as a marine particle-associated, Gram-negative, gammaproteobacterium isolated from the German Wadden Sea. The organism is of interest because of its capability to specifically attach *in vitro* to the surface of the diatom *Thalassiosira weissflogii*-inducing exopolymer and aggregate formation and thus generating marine snow particles [3]. Marine snow formation is an important process of the biological pump, by which atmospheric carbon dioxide is taken up, recycled, and partly exported to the sediments. This sink of organic carbon plays a major role for marine biogeochemical cycles [4]. Several studies reported on the formation and properties of marine aggregates [5-8]. Although it was shown that heterotrophic bacteria control the development and aggregation of marine phytoplankton [3], specific functions of individual bacterial species on diatom aggregation have not been explored thus far.

A better understanding of the molecular basis of bacteria-diatom interactions that lead to marine snow formation is currently gained by establishing a bilateral model system, for which *M. adhaerens* sp. nov. HP15 serves as the bacterial partner of the easy-to-culture diatom, *T. weissflogii* [3]. Herein, we present a set of features for *M. adhaerens* sp. nov. HP15 (**Table 1**) together with its annotated complete genomic sequence, and a detailed analysis of its flagella-associated genes.

**Table 1:** Classification and general features of *M. adhaerens* sp. nov. HP15 according to the MIGS recommendations [9]

**MIGS ID**	**Property**	**Term**	**Evidence code**
		Domain *Bacteria*	TAS [10]
		Phylum *Proteobacteria*	TAS [11]
		Class *Gammaproteobacteria*	TAS [12,13]
	Current classification	Order *Alteromonadales*	TAS [12,14]
		Family *Alteromonadaceae*	TAS [15-17]
		Genus *Marinobacter*	TAS [1,18]
		Species *Marinobacter adhaerens*	TAS [1]
		Type strain HP15	TAS [1]
	Gram stain	negative	IDA
	Cell shape	rod-shaped	IDA
	Motility	motile, single polar flagellum	IDA
	Sporulation	non-sporulating	NAS
	Temperature range	mesophilic	IDA
	Optimum temperature	34-38°C	IDA
	Salinity	0.4-10 g NaCl/l (optimum/growth within 1 day)	IDA
MIGS-22	Oxygen requirement	strictly aerobic	IDA
	Carbon source	dextrin, Tween 40 and 80, pyruvic acid methyl ester, succinic acid mono-methyl-ester, cis-aconitic acid, β-hydroxybutyric acid, γ-hydroxybutyric acid, α-keto glutaric acid, α-keto valeric acid, D,L-lactic acid, bromosuccinic acid, L-alaninamide, D-alanine, L-alanine, L-glutamic acid, L-leucine and L-proline	IDA
	Energy source	chemoorganoheterotrophic	IDA
MIGS-6	Habitat	sea water	IDA
MIGS-15	Biotic relationship	free-living and particle-associated	TAS [2]
MIGS-13	Culture deposition no.	DSM 23420	IDA
MIGS-14	Pathogenicity	none	NAS
	Biosafety level	1	NAS
	Isolation	marine aggregates (0.1-1 mm)	TAS [2]
MIGS-4	Geographic location	German Wadden Sea	TAS [2]
MIGS-4.1	Latitude	53°43’20’’N	TAS [2]
MIGS-4.2	Longitude	07°43’20’’E	TAS [2]
MIGS-4.3	Depth	surface waters	TAS [2]
MIGS-4.4	Altitude	sea level	TAS [2]
MIGS-5	Sample collection time	15 June 2000	TAS [2]

Evidence codes – IDA: inferred from Direct Assay (first time in publication); TAS: Traceable Author Statement (i.e., a direct report exists in the literature); NAS: Non-traceable Author Statement (i.e., not directly observed for the living, isolated sample, but based on a generally accepted property of the species, or anecdotal evidence). These evidence codes are from the Gene Ontology project [19]. If evidence code is IDA, then the property was directly observed for a live isolate by one of the authors or an expert mentioned in the acknowledgements.

## Classification and features

**Figure 1:**
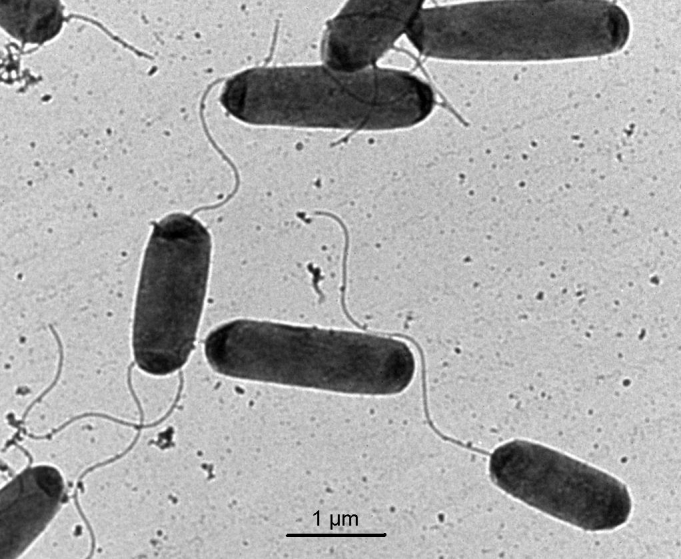
Transmission electron micrograph of *M. adhaerens* sp. nov. strain HP15.

**Figure 2:**
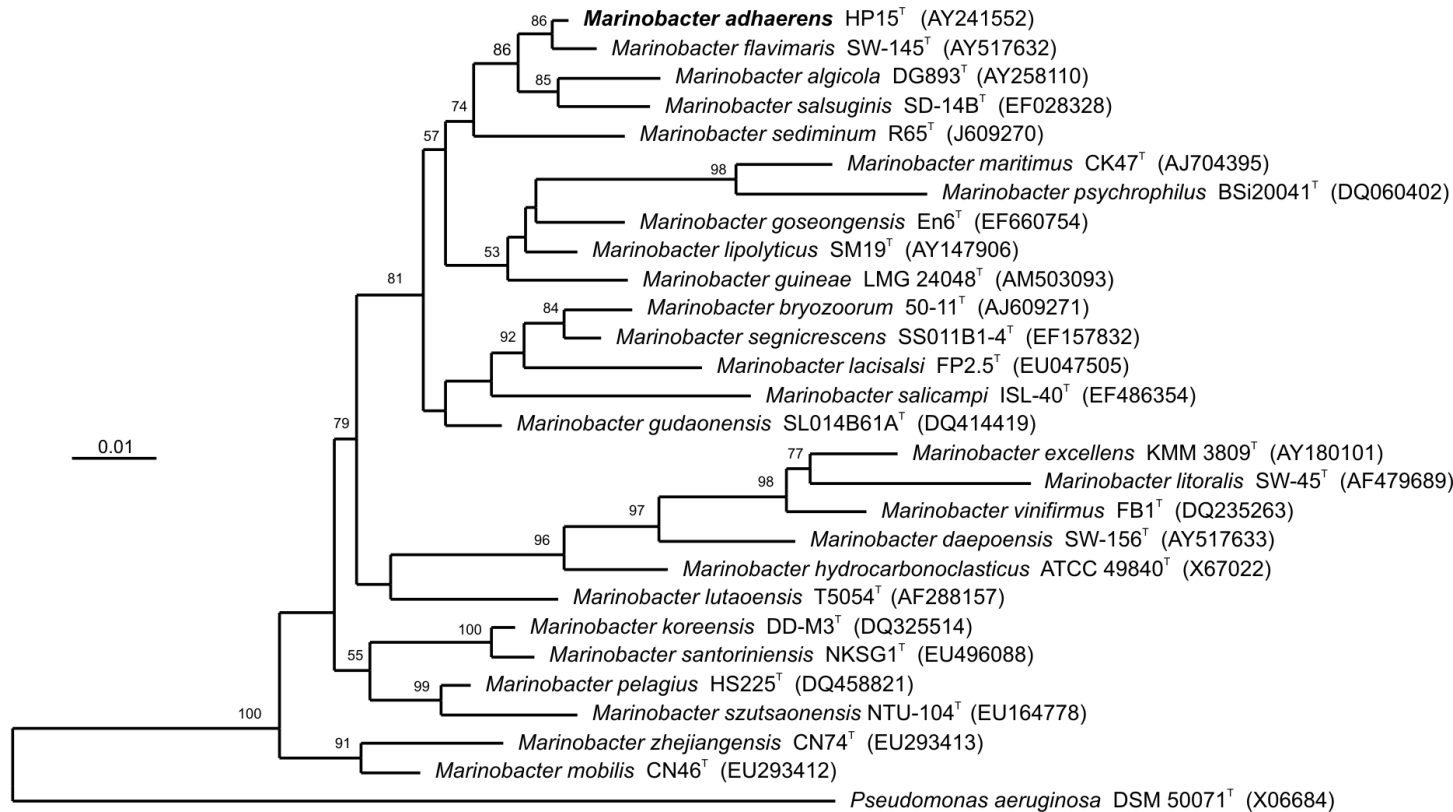
Maximum likelihood phylogenetic tree based on 16S rRNA sequences of *M. adhaerens* type strain (HP15) plus all type strains of the genus *Marinobacter* and the type species of the neighbor order *Pseudomonadales*. Sequence selection and alignment improvements were carried out using the Living Tree Project database [22] and the ARB software package [23]. The tree was inferred from 1,531 alignment positions using RAxML [24] with GTRGAMMA model. Support values from 1,000 bootstrap replicates are displayed above branches if larger than 50%. The scale bar indicates substitutions per site.

*M. adhaerens* sp. nov. strain HP15 is a motile, Gram-negative, non-spore-forming rod (**Figure 1**). Based on its 16S rRNA sequence, strain HP15 was assigned to the *Marinobacter* genus of *Gammaproteobacteria*. Two other *Marinobacter* species were identified based on their interactions with eukaryotes - *M. algicola* isolated from dinoflagellate cultures [20] and *M. bryozoorum* derived from Bryozoa [21]. The 16S rRNA gene of strain HP15 is most closely related to those of the type strains of *M. flavimaris* (99%), *M. salsuginis* (98%) and *M. algicola* (96%). These four type strains form a discrete cluster in the phylogenetic tree (**Figure 2**). In contrast, DNA-DNA hybridization experiments revealed that the genome of *M. adhaerens* sp. nov. HP15 showed about 64% binding to that of *M. flavimaris* [1], which is below the generally accepted species differentiation limit of 70% [25].

### Chemotaxonomy

Strain HP15 can grow in artificial sea water with a nitrogen-to-phosphorus ratio of 15:1 supplemented with glucose as the sole carbon source. In presence of diatom cells but without glucose, HP15 utilized diatom-produced carbohydrates as sole source of carbon. Furthermore, *M. adhaerens* sp. nov. HP15 differed from *M. flavimaris* and other *Marinobacter* species in a number of chemotaxonomic properties, such as utilization of glycerol, fructose, lactic acid, gluconate, alanine, and glutamate [1]. Additionally, strain HP15 showed a unique fatty acid composition pattern.

## Genome sequencing and annotation

### Genome project history

*M. adhaerens* HP15 was selected for sequencing because of its phylogenetic position, its particular feature as a diatom-interacting marine organism [3], and its feasible genetic accessibility to act as a model organism. The respective genome project is deposited in the Genome OnLine Database [19] and the complete genome sequence in GenBank. The main project information is summarized in **Table 2**.

**Table 2:** Genome sequencing project information for *M. adhaerens* sp. nov. HP15

**MIGS ID**	**Property**	**Term**
MIGS-31	Finishing quality	Finished
		
MIGS-28	Library used	454 pyrosequencing standard library
		
MIGS-29	Sequencing platforms	454 FLX Ti
		
MIGS-31.2	Sequencing coverage	22.5× pyrosequencing
		
MIGS-30	Assemblers	Newbler version 2.0.00.22
		
MIGS-32	Gene calling method	GLIMMER v3.02, tRNAScan-SE
		
		CP001978 (chromosome)
	Genbank ID	CP001979 (pHP-42)
		CP001980 (pHP-187)
		
	Genbank Date of Release	September 18, 2010
		
	GOLD ID	Gi06214
		
	NCBI project ID	46089
		
	Database: IMG	pending
		
	Project relevance	Marine diatom-bacteria interactions
		

### Growth conditions and DNA isolation

*M. adhaerens* sp. nov. HP15 was grown in 100 ml Marine Broth medium [26] at 28°C. A total of 23 µg DNA was isolated from the cell paste using a Qiagen DNeasy Blood and Tissue Kit (Qiagen, Hilden, Germany) according to the manufacturer’s instructions.

### Genome sequencing and assembly

The *Marinobacter adhaerens* sp. nov. HP15 genome was sequenced at AGOWA (AGOWA GmbH, Berlin, Germany) using the 454 FLX Ti platform of 454 Life Sciences (Branford, CT, USA). The sequencing library was prepared according to the 454 instructions from genomic *M. adhaerens* sp. nov. HP15 DNA with a final concentration of 153 ng/µl. Sequencing was carried out on a quarter of a 454 picotiterplate, yielding 258.645 reads with an average length of 405 bp, totaling to almost 105 Mb. These reads were assembled using the Newbler assembler version 2.0.00.22 (Roche), resulting in 253.285 fully and 4.763 partially assembled reads, leaving 932 singletons, 226 repeats and 371 outliers. The assembly comprised 112 contigs, with 40 exceeding 500 bp. The latter comprised more than 4.6 Mb, with an average contig size of almost 116 kb and a longest contig of more than 1.2 Mb. Gaps between contigs were closed in a conventional PCR-based gap closure approach, resulting in a fully closed circular chromosome of 4.421.911 bp, and two plasmids of 187.465 bp and 42.349 bp, respectively. Together all sequences provided 22.5× coverage of the genome. The error rate of the completed genome sequence is about 3 in 1,000 (99.7%).

### Genome annotation

Potential protein-coding genes were identified using GLIMMER v3.02 [27], transfer RNA genes were identified using tRNAScan-SE [28] and ribosomal RNA genes were identified via BLAST searches [29] against public nucleotide databases. The annotation of the genome sequence was performed with the GenDB v2.2.1 system [30]. For each predicted gene, similarity searches were performed against public sequence databases (nr, SwissProt, KEGG) and protein family databases (Pfam, InterPro, COG). Signal peptides were predicted with SignalP v3.0 [31,32] and transmembrane helices with TMHMM v2.0 [33]. Based on these observations, annotations were derived in an automated fashion using a fuzzy logic-based approach [34]. Finally, the predictions were manually checked with respect to missing genes in intergenic regions and putative sequencing errors, and the annotations were manually curated using the Artemis 11.3.2 program and refined for each putative gene [35].

## Genome properties

**Figure 3:**
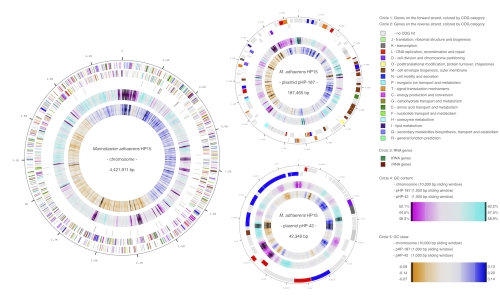
Graphical circular maps of the genome and the two plasmids of HP15. From outside to the center: Genes on forward strand (color by COG categories), Genes on reverse strand (color by COG categories), RNA genes (tRNAs green, rRNAs red, other RNAs black), GC content, GC skew.

The genome of strain HP15 comprises three circular replicons: the 4,422,725 bp chromosome and two plasmids of ~187 kb and ~42 kb, respectively (**Table 3A** and **Figure 3**). The genome possesses a 56.9% GC content (**Table 3B**). Of the 4,482 predicted genes, 4,422 were protein coding genes, and 60 RNAs; 391 pseudogenes were also identified. The majority of the protein-coding genes (67.5%) were assigned with a putative function, while those remaining were annotated as hypothetical proteins. The distribution of genes into COGs functional categories is presented in **Table 4**.

**Table 3A:** Genome composition for *M. adhaerens* HP15

**Label**	**Size (Mb)**	**Topology**	**RefSeq ID**
Chromosome**^§^**	4.423	circular	CP001978
Plasmid pHP-187**^¶^**	0.187	circular	CP001980
Plasmid pHP-42*	0.042	circular	CP001979

**^§^** Number of protein-coding genes: 4,180; ^¶^ Number of protein-coding genes: 178;

* Number of protein-coding genes: 52

**Table 3B:** Genome statistics for *M. adhaerens* HP15

**Attribute**	**Value**	**% of total^a^**
Genome size (bp)	4,651,725	
DNA Coding region (bp)	4,178,502	89.8
DNA G+C content (bp)	2,644,970	56.9
Number of replicons	3	
Extrachromosomal elements	2	
Total genes^b^	4,410	
tRNA genes	51	1.16
5S rRNA genes	3	0.07
16S rRNA genes	3	0.07
23S rRNA genes	3	0.07
Protein-coding genes	4,355	98.66
Genes assigned to COGs	3,027	67.54
Genes with Pfam domains	2,918	65.1
1 Pfam domain	2,041	45.54
2 Pfam domains	598	13.34
3 Pfam domains	194	4.33
4 or more Pfam domains	85	1.9
Genes with signal peptides	765	17.07
Genes with transmembrane helices	1,043	23.27
1 transmembrane helix	341	7.61
2 transmembrane helices	154	3.44
3 transmembrane helices	72	1.61
4 or more transmembrane helices	476	10.62
Genes in paralogous clusters	570	12.72
Genes with 1 paralog	364	8.12
Genes with 2 paralogs	63	1.41
Genes with 3 paralogs	26	0.58
Genes with 4 or more paralogs	117	2.61
Pseudo/hypothetical genes	391	8.72
Conserved hypothetical genes	668	14.90
Genes for function prediction	3,363	75.03

a) The total is based on either the size of the genome in base pairs or the total number of protein coding genes in the annotated genome.

b) Also includes 54 pseudogenes and 5 other genes.

**Table 4:** Number of genes associated with the 21 general COG functional categories

**Code**	**Value**	**%age^a^**	**Description**
J	162	3.7	Translation
A	0	0	RNA processing and modification
K	161	3.6	Transcription
L	132	3	Replication, recombination and repair
B	0	0	Chromatin structure and dynamics
D	32	0.7	Cell cycle control, mitosis and meiosis
Y	0	0	Nuclear structure
V	0	0	Defense mechanisms
T	199	4.5	Signal transduction mechanisms
M	151	3.4	Cell wall/membrane biogenesis
N	166	3.8	Cell motility
Z	0	0	Cytoskeleton
W	0	0	Extracellular structures
U	0	0	Intracellular trafficking and secretion
O	127	2.9	Posttranslational modification, protein turnover, chaperones
C	192	4.3	Energy production and conversion
G	82	1.9	Carbohydrate transport and metabolism
E	254	5.7	Amino acid transport and metabolism
F	51	1.1	Nucleotide transport and metabolism
H	97	2.2	Coenzyme transport and metabolism
I	141	3.2	Lipid transport and metabolism
P	138	3.1	Inorganic ion transport and metabolism
Q	76	1.7	Secondary metabolites biosynthesis, transport and catabolism
R	330	7.5	General function prediction only
S	251	5.7	Function unknown
-	285	6.4	multiple COGs
	3,027	68.6	Total
-	1,383	31.4	Not in COGs

a) The total is based on the total number of protein coding genes in the annotated genome

## Flagella-associated gene clusters of *M. adhaerens* HP15

**Figure 4:**
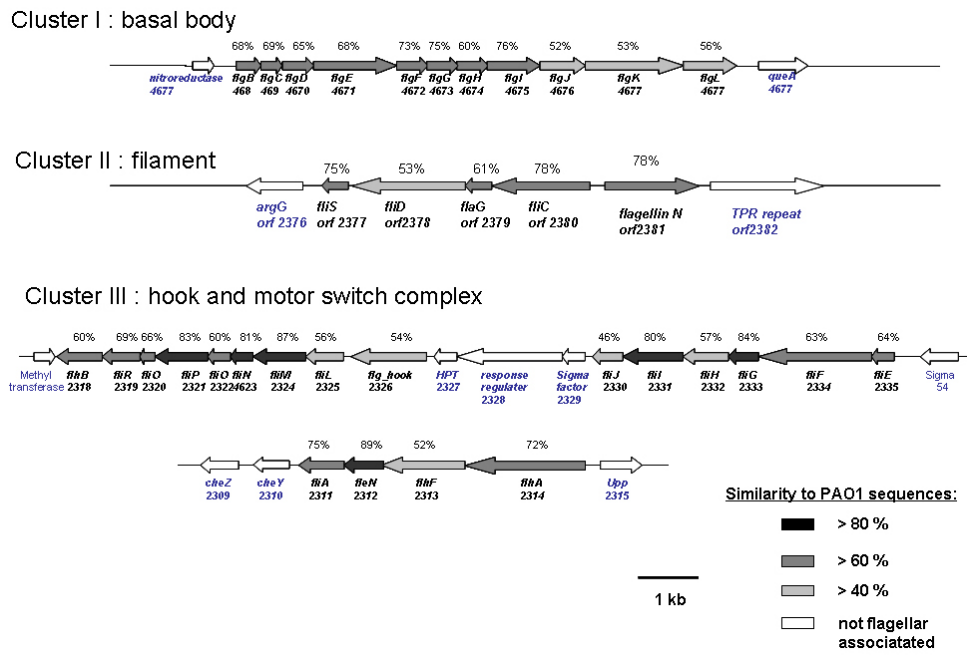
Schematic presentation of the three flagella-associated gene clusters of *M. adhaerens* HP15 coding for the basal body, the filament, and the hook and motor switch complex. Identities to the respective orthologs in the genome of *P. aeruginosa* PAO1 are indicated by gray-scale code. Numbers of CDS are shown below gene names.

Because *M. adhaerens* HP15 was experimentally shown to adhere to diatom cells, gene clusters coding for secretion, assembly, and mechanistic function of the polar flagellum were analyzed in detail (**Figure 4**). Besides several other chemotactic mechanisms and various cell surface interactions, bacterial flagella and other cell appendages had previously been shown to be instrumental for chemotactic movement towards and adhesion to biotic surfaces [36,37]. The amino acid sequences of proteins encoded by the three identified gene clusters showed significant similarities to orthologous and experimentally well-described gene products of *P. aeruginosa* PAO1 and various other bacterial species as determined by BLASTP algorithm comparison using the Blosum 62 substitution matrix [29]. Not surprisingly, hook and motor switch complex components were most conserved. However, gene products involved in flagellar filament formation encoded by Cluster II also showed 53 to 78% similarity to the respective PAO1 proteins. Mutagenesis of flagella-associated genes of *M. adhaerens* HP15 will be carried out in the near future to study the role of flagella in bacteria-diatom interactions and to further our understanding of the cell-to-cell communication between those organisms.
